# Maintenance of dental records and awareness of forensic odontology among pakistani dentists: a mixed-method study with implications for dental data repository

**DOI:** 10.1186/s12903-023-03500-2

**Published:** 2023-10-24

**Authors:** Haifa Saquib Baqai, Syed Jaffar Abbas Zaidi, Qaiser Ali Baig, Muhammad Bilal Bashir, Madiha Anwar, Asma Saher Ansari

**Affiliations:** 1 Department of Oral Biology, Hamdard Dental College, Hamdard University, Karachi, Pakistan; 2 Department of Oral Biology, Dow Dental College, Dow University of Health Sciences, Karachi, Sindh 74200 Pakistan; 3 Department of Community Dentistry, Dow International Dental College, Dow University of Health Sciences, Karachi, Pakistan; 4 Department of Oral Biology, Fatima Jinnah Dental College, Karachi, Pakistan; 5 Department of Oral Biology, Bahria University Medical & Dental College, Karachi, Pakistan; 6 Department of Oral Biology, Dr Ishrat ul Ebad Khan Institute of Oral Health Sciences, Dow University of Health Sciences, Karachi, Pakistan

**Keywords:** Forensic odontology, Mixed method study, Dental records, Repository

## Abstract

**Objective:**

Dental records and forensic odontology play an important role in both healthcare and the legal system, aiding in personalized patient care, human identification, and legal proceedings. This study aims to investigate dental record-keeping practices and assess the awareness of forensic odontology among Pakistani dentists over 12 months. This study aims to collect data from 500 dentists, identify areas for enhancement, and develop a strategic action plan to improve record-keeping quality and forensic odontology application, culminating in a comprehensive dental data repository to support legal and criminal investigations in Pakistan.

**Methodology:**

This study employed a mixed-method approach conducted at Hamdard Dental College from January to March 2023. The quantitative phase involved distributing questionnaires to 463 dentists, chosen through stratified random sampling. Out of these, 413 responded, yielding an 86% response rate. These questionnaires focused on dental record-keeping practices and dentists’ awareness of forensic odontology. Subsequently, based on the questionnaire results, face-to-face interviews were conducted with 20 purposively selected dentists to gain deeper insight into the challenges and potential solutions. Data from both phases were integrated and analyzed accordingly.

**Results:**

The study included 413 participants, mainly females (79%), with ages ranging from 27 to 65 years and an average age of 46.4 years. Most dentists had 5–20 years of work experience (53%), and most (87.4%) were practicing in private clinical settings. All the dentists generated medical and dental records, but the duration of their record-keeping varied, with some maintaining them for up to a year and others for two years or longer. Five themes were generated from the qualitative content analysis. These themes were dentists’ perceptions, barriers and challenges, knowledge and awareness, and improvement strategies.

**Conclusion:**

Our study revealed that local practitioners in Pakistan exhibit subpar practices in dental record-keeping and maintenance of patient history, irrespective of whether they use a digital or traditional file-based system. Even though dentists are cognizant of the importance of record-keeping, they do not actively maintain comprehensive records. This suggests the need for improved training and system improvements to address the gaps in record-keeping practices.

## Introduction

Forensic odontology is a compelling and challenging field within forensic science, enabling the recognition of deceased individuals by comparing their pre- and postmortem dental records [1]. The significance of dental identification methods, used since AD 66, was first legally acknowledged in 1849 when dental evidence was accepted in a legal setting.

Forensic odontology has grown over the past hundred years to become a pivotal aspect of forensic medicine, affirming its position as an essential component in medico-legal scenarios. Today, it stands as one of the fastest-growing sectors within the realm of dental science. Its significance becomes even more pronounced in circumstances involving mass tragedies such as internal conflicts, terror attacks, or acts of genocide, where the victims’ bodies could be severely disfigured or incinerated.

The multifaceted nature is encapsulated in the Avon classification system, which divides it into three main sectors: civil cases, criminal investigations, and research initiatives [2]. This scientific discipline spans a wide range of specializations, including examining bite mark injuries, probing instances of child maltreatment, and identifying human remains in situations of mass casualties. It also delves into specific areas such as cheiloscopy, examination of lip impressions, and rugoscopy, which involves the study of patterns on the palate rugae.

Snyder proposed the potential of lip patterns as unique identification markers akin to fingerprints in 1950, later modified by Sukuki et al. [3]. They demonstrated that even monozygotic twins exhibit distinct lip patterns. Historically, the temporal stability of lip prints was ambiguous. Emerging evidence elucidates this aspect; however, its clarity was initially lacking. Lip rugae develop when the embryonic length reaches approximately 5.5 cm, with prominence during mid-age and eventual regression [4].

Palatal rugae exhibit significant value in forensic dentistry due to their distinctive nature and resilience to postmortem changes, making them vital for human identification [5]. The uniqueness and stability of these rugae patterns present an exceptional tool for individual identification, enhancing the repertoire of forensic odontology [6]. However, some researchers have highlighted that they tend to diminish in number with increasing age and may be impacted by orthodontic treatment [7]. The classification of rugae remains a complex task, requiring substantial additional research [8].

The identification of human remains traditionally relies on personal artifacts such as clothing, jewelry, fingerprints, blood groups, and dental records. The emerging field of forensic odontology also utilizes sialochemistry, or the detection of salivary chemicals, as a potential investigative tool.

Dental record-keeping and forensic odontology are pivotal in healthcare and criminal justice. Dental records, encompassing patient identification, treatment plans, medical history, and radiographs, are invaluable for providing personalized patient care and maintaining continuity in treatment approaches [9]. Even without such records, a forensic odontologist possesses the expertise to identify deceased individuals utilizing distinctive dental characteristics. Simultaneously, in forensic odontology, these records play a crucial part in human identification in mass disasters, age estimation, bite mark analysis, and cases of abuse and malpractice litigation. Dental practitioners must comprehend the legal ramifications of these situations, particularly in the contemporary context marked by an increased frequency of natural and anthropogenic disasters and a surge in criminal activities, including heinous acts such as gang rape and child abuse [10]. Notably, under such circumstances, the remains are often mutilated to a degree that impedes visual recognition [11].

Although a well-established discipline in several parts of the world, forensic odontology has yet to achieve the same recognition in Pakistan [12]. Several factors contribute to this gap, including limited awareness about the significance of the field, the absence of a standardized protocol for maintaining dental records, and the lack of a centralized dental data repository.

In Pakistan, dentistry has been evolving rapidly over the last few decades, with an increasing number of dental schools and professionals entering the field. However, research on dental record maintenance and the understanding and application of forensic odontology among these professionals remains scant. This lack of data hinders the development of strategies to advance these critical areas.

This study aims to specifically investigate the current practices of dental record-keeping and understand the awareness level of forensic odontology among Pakistani dentists within a 12-month timeframe. By utilizing a measurable validated survey and qualitative assessment tools, we intend to gather data from a sample size of 500 dentists across the country. The achievable goal is to highlight areas of improvement and based on the findings, realistically develop a strategic action plan to enhance the quality of dental record-keeping and bolster the application of forensic odontology. This will timely lead to the establishment of a comprehensive dental data repository and increased contributions of the dental field to legal and criminal investigations in Pakistan.

## Methodology

From January to March 2023, a mixed-methods approach was used to ensure a comprehensive understanding of the subject matter through a quantitative online survey and qualitative face-to-face interviews. For the quantitative portion of the study, a questionnaire was developed after an extensive review of the relevant literature. This 14-item questionnaire was designed to gather information on dentists’ practices regarding dental record-keeping and their awareness and perceptions of forensic odontology over a period of 12 months. The questions ranged from record-keeping habits, age estimation methods, recognition of child abuse signs, and potential barriers in maintaining records. The participants’ responses to this questionnaire provided a comprehensive understanding of their involvement and challenges in forensic odontology, laying a substantial groundwork for the findings of our study. This questionnaire was pretested on a small sample for clarity and comprehensibility before being finalized. The inclusion criteria in this study encompassed dentists working in both public and private sector practice, including hospital settings. Conversely, dentists not currently in practice were excluded from the study.

The questionnaire was disseminated through Google Forms to 500 practicing dentists across Pakistan, selected through a stratified random sampling method, ensuring a representative sample of the dentist population. Out of 500 dentists, 413 completed and returned the questionnaire, giving a response rate of approximately 82%. Statistics were calculated by applying the chi-square test in SPSS 26.0.

Following the survey, in-depth face-to-face interviews were conducted with 20 dentists. The participants were purposively selected based on their questionnaire responses. Specifically, those who indicated “strongly agree” and reported a high level of awareness about forensic odontology were chosen. In contrast, those who selected “strongly disagree” and demonstrated a lower awareness of forensic odontology were also included. This selection criterion was employed to capture and explore both extreme views on the topic. The interview guide was developed with open-ended questions to comprehensively investigate the barriers, perceptions, and potential solutions for the issues highlighted in the questionnaire. All interviews took place in a quiet and private setting, were digitally recorded, and subsequently transcribed verbatim for analysis. Each interview, lasting 45 min to an hour, was conducted by a single investigator. Data from both the questionnaire and interview phases were integrated and analyzed to formulate conclusions and recommendations for enhancing dental record-keeping and the application of forensic odontology in Pakistan.

The methodology for the qualitative study was guided by Braun and Clarke’s six-step approach to thematic analysis, a commonly employed qualitative research method [13]. These codes were then organized into potential themes, where related codes were consolidated based on their shared meaning or content. For instance, codes concerning ‘record maintenance’ and ‘record-keeping duration’ were assembled under the overarching theme of ‘Record Keeping Practices.‘

The following step entailed a rigorous review and refinement of these themes. This process consisted of two levels: evaluation at the level of coded data and at the level of the complete data set. The goal was to ensure that the themes corresponded to the codes within them and accurately reflected the entire data set. Subsequently, the themes were named and defined. Each theme was assigned a clear, concise name that encapsulated the core of what the theme represented. Simultaneously, clear outlines of each theme’s scope and content were defined.

The final phase involved analyzing the themes concerning the research objectives. This involved probing into how each theme contributed to understanding the research objectives and providing answers to the research questions. Throughout this analytical process, the principle of reflexivity was upheld [14]. The authors introspectively considered potential biases and how these might influence the data analysis, thereby ensuring the integrity and validity of the process. The themes and categories with their representative sentences are shown in Table 1.

**Table 1:** Codes and themes related to forensic odontology

Theme	Category	Code	Representative Sentences
Dentists’ Perceptions of FO	Importance of Dental Records	FDR1	“I consider the maintenance of dental records as a crucial part of my practice.“
	Practice of Maintaining Records	FDR2	“In my practice, I adhere to a specific routine for maintaining dental records to avoid litigations.“
Influential Factors	Duration of Record-Keeping	FRK1	“Various factors, such as the number of patients I see and storage limitations, influence how long I keep dental records.“
Barriers and Challenges	Implementation of Record Keeping	FBC3	“When trying to implement regular and effective dental record-keeping practices, I face challenges like limited resources and time constraints.“
	Digital record-keeping	FBC4	“I am using digital record-keeping apps provided by https://oladoc.com/ and www.marham.pk. It has made my life easy, and they refer patients too.”
Knowledge and Awareness of dental record-keeping	Use of Dental Records in Forensic Odontology	FDR4	“I only keep X-rays and Dental OPGs of my patients for record-keeping purposes.“
		FDR5	“The most comprehensive records are of my orthodontic patients, which I keep for the duration of their treatment. This on average being for two years”.
Improvement Strategies	Enhancing Record Keeping and Forensic Use	FIR5	“I believe strategies like conducting training workshops and digitalizing records can enhance dental record-keeping practices, but stricter laws are needed for its universal implementation.“

## Results

This study involved a total of 413 participants, comprising n = 326 (79%) females and n = 87 (21%) males. The age range of the respondents spanned from 27 to 65 years, with a mean age of 46.4 years (+ 4.5 SD). Participants’ professional experience was categorized as follows: n = 95 (23%) had less than five years of experience, n = 219 (53%) had between five to twenty years, and n = 99 (24%) had over twenty years of experience. Most dental surgeons, n = 361 (87.4%), were engaged in private clinical practice, while n = 52 (12.6%) were associated with the public sector.

The study’s findings revealed that general dental practitioners and authorities engage in record maintenance and record-keeping, as shown in Table 2. All 413 dental surgeons confirmed the generation of medical and dental records. However, the duration of record retention varied, with some retaining records for up to a year and others for longer periods, extending beyond two years.

**Table 2:** Maintenance and pattern of storage of dental records, awareness of medico-legal cases

	Value	Response (n = 413)	Response in Percentage	p-value
Duration of Maintenance of Records	0 to 6 months	107	26%	0.05
	6 months to 1 year	132	32%	
	1 to 2 years	29	7%	
	2 years onwards	144	35%	
Pattern of Storage of Records	Manual	157	38%	0.003
	Computerized	58	14%	
	Both	198	48%	
Awareness of Child Abuse	Yes	355	86%	0.009
	No	58	14.0%	

**Fig. 1:**
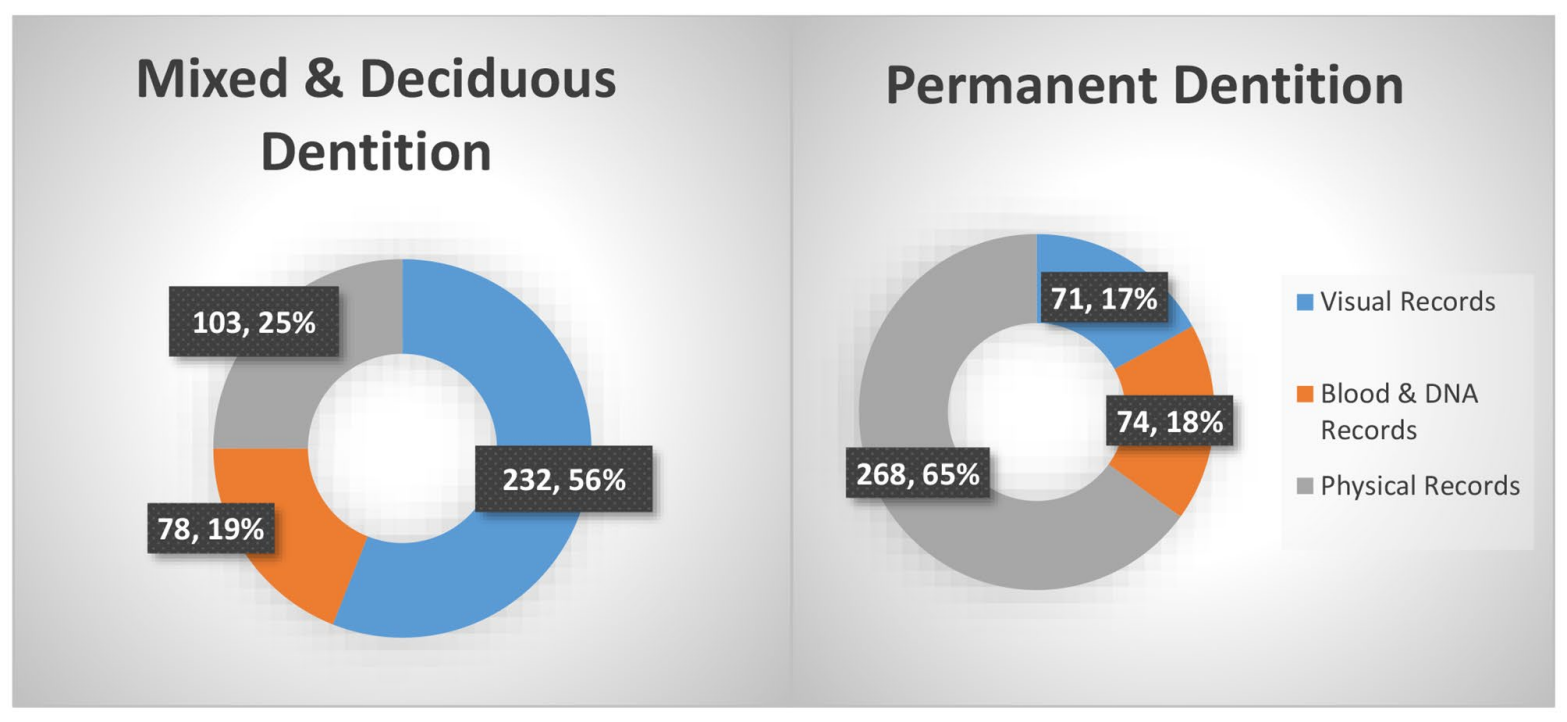
Knowledge of Age Estimation from Records of Mixed, Deciduous & Permanent Dentition

Surveyed dentists were queried regarding their proficiency in estimating a subject’s age using dental records. The most prevalent method cited involved visually assessing the subject’s age by inspecting the primary and mixed dentition. For permanent teeth, age approximation primarily relied on physical documentation, while estimating age and identifying subjects using biochemical or DNA-based records presented challenges to clinicians in both types of dentitions (Fig. 1).

The records maintained in dental clinical settings encompassed a broad spectrum, including patient personal data, photographs, medical and family histories, dental casts, radiographs, treatment logs, plans, investigations reports, and clinical findings. The frequency of these record-keeping practices, evaluated on a Likert scale of ‘always,‘ ‘often,‘ ‘sometimes,‘ and ‘rarely,‘ is delineated in Table 3.

**Table 3:** Frequency of the type of record maintained in dental clinics

Frequency	PATIENT DETAILS n (%)	PHOTOGRAPHS n (%)	MEDICAL HISTORY n (%)	FAMILY HISTORY n (%)	DENTAL CASTS n (%)
Always	355 (86)	95 (23)	54(13)	58(14)	99(24)
Often	58 (14)	198 (48)	45(11)	120(29)	153(37)
Sometimes	0	45 (11)	314(76)	235(57)	132(32)
Rarely	0	75 (18)	0	0	29(7)
	**RADIOGRAPHS** **n (%)**	**TREATMENT PLAN** **n (%)**	**INVESTIGATION REPORTS** **n (%)**	**TREATMENT LOG** **n (%)**	**CLINICAL FINDINGS** **n (%)**
Always	285 (69)	157 (38)	74 (18)	140 (34)	70 (17)
Often	95 (23)	107 (26)	67 (16)	161 (39)	99 (24)
Sometimes	33 (8)	149 (36)	272 (66)	112 (27)	186 (45)
Rarely	0	0	0	0	58 (14)

## Discussion

Forensic odontology is fundamentally about deciphering complex cases of abuse and fatalities using dental expertise. Dental practitioners need to be knowledgeable and aware of FO [15]. Historical documents sporadically reference the utilization of dental identification, implying that rudimentary versions of this practice could date back to prehistoric eras [8].

The earliest documented instance of dental identification is traced back to the period between 49 and 66 AD, during the reign of the Roman Emperor Claudius. Agrippina, Claudius’s wife, allegedly fueled by jealousy, demanded the examination of her husband’s deceased mistress, Lollia Paulina. The identification of the deceased was confirmed based on a discolored anterior tooth and malocclusion [16].

In a study by Acharya AB in 2006, a survey was conducted across five universities, revealing that these institutions possessed an in-depth curriculum dedicated to forensic dentistry and adhered to a standardized teaching protocol for the discipline [12]. Nevertheless, it was inferred that the curriculum might not fully include the most recent advancements in forensic dentistry.

The role of dental records and forensic odontology in healthcare and legal systems is well-recognized worldwide. In essence, dental records serve as an essential tool in individualized patient care and human identification, especially in mass disasters and in various legal matters, such as age estimation and bite mark analysis [9].

The present study’s finding, wherein most participants exhibited subpar practices in dental record-keeping, resonates with previous research from other developing countries. A study in Australia reported a lack of uniformity in dental record-keeping, indicating a dire need for standardized protocols [17]. However, the high rate of response (86%) from the participants in this study accentuates the significance of this issue among Pakistani dentists and underscores the gravity with which they view this problem.

In our sample of 413 participating dental professionals, we observed a female preponderance with a gender distribution of 79% females (n = 326) and 21% males (n = 87), echoing trends within the dental community. Age ranged from a youthful 27 to a seasoned 65, with an average age of 46.4 years (+ 4.5 SD). Notably, our sample encompassed a wide array of experience levels, from recent entrants with less than five years of experience (n = 95, 23%) to veterans boasting over two decades in the field (n = 99, 24%).

An apparent inclination towards private practice was detected, with 87.4% of respondents (n = 361) operating within private clinical environments, as opposed to a mere 12.6% (n = 52) practicing within public sectors. This disparity might suggest a preference for autonomy and potential financial advantages associated with private practice.

Turning to record-keeping practices, we identified a universal trend among the surveyed dentists: all maintained medical and dental records, as shown in Table 2. The durations of record retention varied, reflecting either short-term storage of up to a year or longer-term archiving spanning two years or more.

Most respondents favored visual examination of deciduous teeth when asked about their ability to estimate patient ages from medical records. The permanent dentition identification process leaned heavily towards physical records, while biochemical/DNA-based records presented a challenge for both types of dentitions (Fig. 1).

A wide array of records was maintained within dental clinical setups, ranging from personal patient information to clinical findings. Their regularity of maintenance gauged on a Likert scale, varied across the different types of records (Table 3). These findings are in agreement with the record-keeping practices of dentists observed in various countries [18, 19].

These findings underline the perceived significance of record maintenance among dental practitioners and highlight the diverse types of records that indicate a comprehensive approach to documentation, as shown by a study conducted by Kashif et al. [20]. Still, specific challenges persist, such as interpreting biochemical/DNA-based records [21].

This study provides crucial insights into the current state of dental record-keeping among Pakistani dentists, broadly consistent with previous research in this field. Dentists’ awareness and understanding of the importance of comprehensive record-keeping practices align with Del Rose et al. (2000) findings, which underscore the dental community’s recognition of the critical role these records play in patient management and medico-legal contexts [22]. Similarly, a systematic review of awareness of forensic odontology among dental professionals in Saudi Arabia revealed similar results [23].

Interestingly, our findings show a strong leaning towards private practice, which dovetails with the global trend of the increasing preference for private over public practice due to associated autonomy and financial benefits [24].

As observed in this study, the extensive variety of records maintained in dental clinics also aligns with the American Dental Association’s (ADA) recommendations, emphasizing the necessity of thorough documentation for quality patient care [22]. However, the challenge noted in our study regarding the interpretation of biochemical/DNA-based records is a relatively less explored area in the literature [25]. It signals an opportunity for further research and training initiatives.

Our findings about the variation in the duration of record maintenance echo the study of Chandra Shekar and Reddy (2011), who noted that factors like storage space and the number of patients often influence the duration for which records are maintained [1].

In summary, the results of this study broadly concur with the current literature, underscoring the universal importance attributed to record-keeping in dental practice while highlighting the need for further research and training to navigate the challenges posed by the increasing role of advanced biochemical and genetic markers in dental record-keeping [22].

In Pakistan, law enforcement agencies typically consult dental surgeons affiliated with government services rather than experienced and certified forensic odontologists in private practice. This preference cultivates a perception of an incomplete professional scope among dental practitioners, discouraging them from entering this specialized field. Under adverse circumstances, forensic dentistry can play an essential role in identifying deceased or living individuals [26].

One reason for this problem is that forensic odontology is not taught as a separate subject in Pakistani educational institutions. As stipulated in the Bachelor of Dental Surgery (BDS) curriculum approved by the regulatory body of dental schools in Pakistan, a mere four hours of teaching was allocated to this subject, integrated into the primary oral surgery curriculum [27].

Historical evidence suggests the potential significance of forensic odontology in solving significant cases. For instance, the late President of Pakistan, General Zia-ul-Haq, tragically perished in a plane explosion in 1988 and was identified posthumously through his dental records [28]. Similarly, the late Indian Prime Minister, Mr. Rajiv Gandhi, a victim of a terrorist attack in 1991, was also identified through his dentition [29].

It has been shown that teeth can be used as defensive and offensive weapons, and bite marks can reveal a person’s identity [30]. Since most abuse injuries occur in the head and neck, dentists can quickly diagnose them. Teeth can inflict serious injury on an attacker and may be the only defensive method for the victim. Alternatively, it is well known that assailants in sexual attacks, including sexual homicide, rape, and child sexual abuse, often bite their victims as an expression of dominance, rage, and animalistic behavior. In the current study, only 86% of dental practitioners were unaware of how to identify child abuse, and only a handful knew how to deal with a child abuse case. According to a study by Namrata et al., 61% of dental practitioners in India were unaware of child abuse [31]. In contrast, according to a study by Preethi et al., 40% of dental practitioners were unable to recognize child maltreatment [32].

Research participants in this study were asked to express their proficiency in deducing a person’s age through dental data. The principal method highlighted was the visual evaluation of primary and mixed teeth to determine the age. The reliability of age estimation during juvenile age has been documented in the literature [33]. For permanent teeth, age estimation was predominantly based on visual inspection of the dentition, while age approximation and identification using biochemical or genetic records presented complications for both sets of teeth [34].

The findings of this survey mirror those reported in the literature. According to Namene et al., most professionals are comfortable estimating age through visual examination of primary and mixed dentition, reaffirming the results of this survey [35]. Similarly, Schemling et al. found that age approximation in permanent teeth relied heavily on physical records, consistent with our findings [36]. A recent review also highlighted the importance of age estimation and its non-invasive nature through dental records [37].

Contrarily, Malik et al. (2022) reported that using biochemical or genetic records for age estimation was gaining acceptance among professionals, conflicting with our survey findings where the participants expressed difficulties using these records [38]. This discrepancy may be due to varying levels of access to and proficiency with advanced biochemical and genetic techniques among professionals. However, additional research is needed to verify and elaborate on these contrasting findings. Future studies could examine the barriers and difficulties professionals face when using biochemical or DNA-based records for age estimation.

Age determination serves numerous purposes, such as determining the age of a corpse in a criminal case or a severely damaged body after a natural disaster [39]. Age estimation can nullify false age claims to avoid prison sentences. Furthermore, determining age is often required for criminal acts like rape and kidnapping, and falsifying age can also be seen in employment, immigration, and marriages [40]. If a birth certificate is unavailable or a record is doubtful, age determination can be an extremely valuable tool for the justice system [41].

**Fig. 2:**
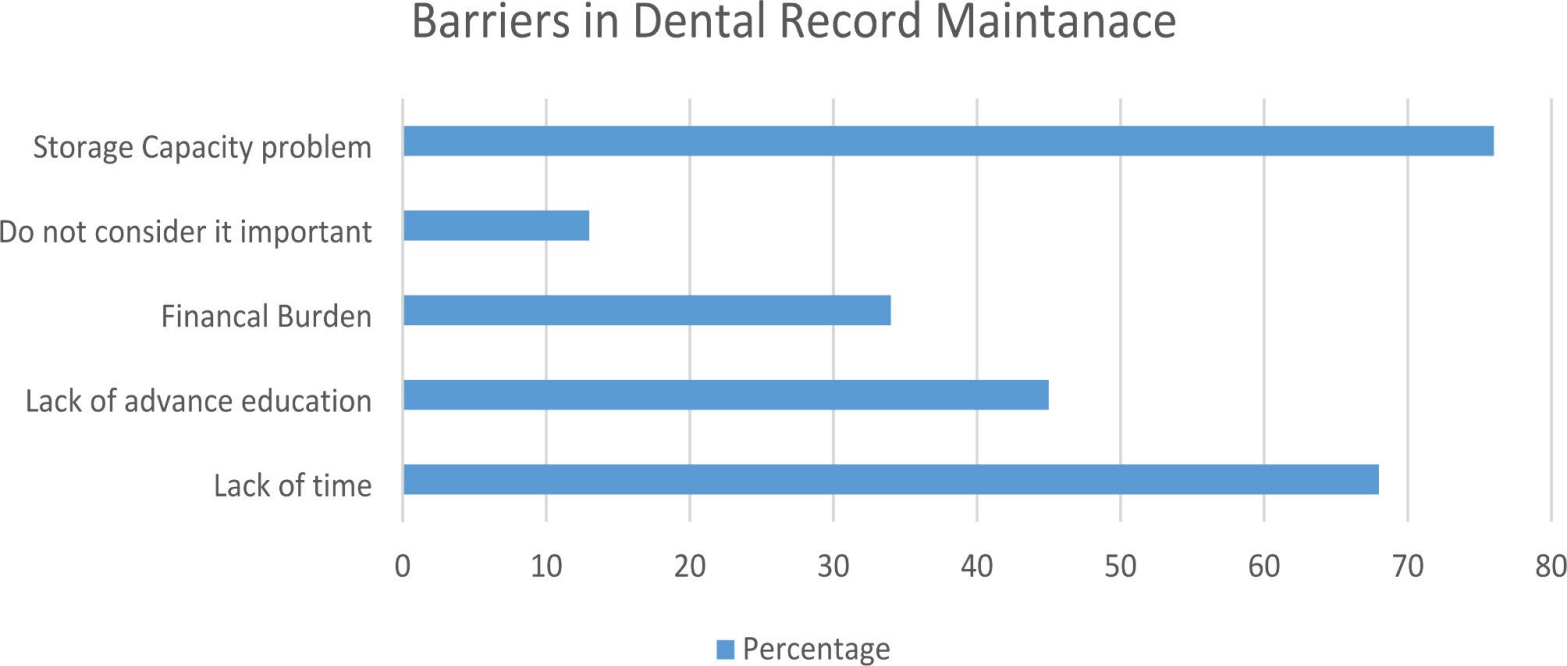
Reasons that cause hindrance in dental record maintenance

In the present study, several obstacles were identified as impediments to maintaining dental records, as shown in Fig. 2. These encompassed limitations in storage capacity, perceptions of record keeping being unimportant, financial constraints, time restrictions, and insufficiently advanced education on the topic. These findings are in agreement with previous research [42].

Identifying many causalities in a mass disaster is complex and fraught with physical and emotional hazards. Dental identification is a cost-effective, rapid, and non-invasive method for developing countries such as Pakistan to consider in disaster victim identification. Alterations due to toothburn have been extensively documented in the literature [43, 44]. These studies have shown that even minute teeth fragments can be identified amongst burn residues [38]. Moreover, they have provided a reliable methodology to estimate the exposure temperature, demonstrating that there may be no or minimal anatomical changes from such exposure.

The qualitative content analysis further sheds light on the underlying reasons for these practices. The identified themes, such as barriers and challenges, knowledge and awareness, and improvement strategies, align well with findings from similar studies in other regions [10, 45]. Particularly, the theme of “dentists’ perceptions” might be a keystone in understanding the prevailing practices. Dentists’ perceptions, molded by their cultural, educational, and societal background, can significantly influence their record-keeping behavior [37].

Five themes were generated from the qualitative content analysis. These themes were dentists’ perceptions, barriers and challenges, knowledge and awareness, and improvement strategies.

### Dentists’ perceptions of forensic odontology

Two categories were identified under the theme of dentists’ perceptions of FO. These were the importance of dental records and the practice of maintaining records.

The dentists interviewed stressed that the dental record is an established practice of private practitioners. However, not all dental records are kept. Some dentists keep dental records while providing dental treatment and discard them after treatment.

In a comprehensive patient care setting, medico-legal matters, and research, dental records provide accurate information about treatments, diagnoses, and patients’ specific dental conditions. The maintenance of meticulous dental records improves patient care and improves dental audits, quality assurance, and overall administrative processes.

“I consider the maintenance of dental records as a crucial part of my practice.“ (FDR1).

“In my practice, I adhere to a specific routine for maintaining dental records to avoid litigations.“ (FDR2).

### Influential factors

“Duration of Record Keeping” was the category under the overarching “Influential factors” theme.

The statement reflects a common concern in health record management regarding the duration of retaining patient records, which numerous factors like patient load and physical or digital storage capacity can influence.

The number of patients a practitioner sees can impact record retention. High patient turnover can lead to an increased volume of records, potentially exceeding storage capabilities and necessitating a more limited retention period. Furthermore, legislative requirements vary by country and state, and these often stipulate a minimum duration for which records must be kept [46]. These can range from a few years to several decades. However, in Pakistan, there are no legal requirements for maintaining dental records. Overall, while the optimal scenario is to support comprehensive, lifelong patient records, practical constraints such as the volume of patients and storage limitations often dictate the retention period of these records [47].

“Various factors, such as the number of patients I see and storage limitations, influence how long I keep dental records.“ (FRK1).

### Barriers and challenges

The category identified in this theme was the implementation of record keeping and digital record keeping. There were two conflicting scenarios on this theme. Some older dentists faced issues in record keeping, such as limited resources and staff, while younger dentists increasingly used digital apps for record keeping. These statements reflect the increasing transition trend from traditional to digital methods in dental record-keeping, a topic extensively discussed in health informatics literature [48]. This reflects the literature suggesting that the adoption of electronic health records (EHRs) or digital platforms can improve efficiency and accessibility and lead to new patient referrals through networked systems. The digital apps facilitate online appointments, digital record keeping, and a network of healthcare professionals for referrals [49].

“When trying to implement regular and effective dental record-keeping practices, I face challenges like limited resources and time constraints.“ (FBC3).

“I am using digital record keeping apps provided by https://oladoc.com/ and www.marham.pk. It has made my life easy, and they refer patients too” (FBC4).

### Knowledge and awareness of dental record keeping

The category identified under this theme was “Use of Dental Records in Forensic Odontology.”

“I only keep X-rays and Dental OPGs of my patients for record-keeping purposes.“ (FDR4).

Retaining radiographs as primary components of patient records aligns with the statutory requirements of many countries as it emphasizes the crucial role radiographs play in diagnosis, treatment planning, and monitoring [45]. However, this approach seems to overlook the importance of holistic record-keeping, which includes but is not limited to patient histories, clinical notes, and correspondence with other clinicians, factors that could provide a more comprehensive view of the patient’s dental and overall health. This lack of dental record-keeping was also reported by a recent systematic review conducted in India [50].

“The most comprehensive records are of my orthodontic patients, which I keep for the duration of their treatment. This on average being for two years.” (FDR5).

The practice of retaining detailed records of orthodontic patients for the duration of their treatment, typically around two years, matches industry norms for orthodontic treatments and is in line with contemporary record-keeping practices during active therapies [42]. However, it raises questions regarding the maintenance of records post-treatment. Retaining records for a stipulated period after treatment is essential for medico-legal purposes, future therapies, and longitudinal studies of orthodontic outcomes [51]. Therefore, although the practice is suitable for ongoing treatment, a review of the retention of post-treatment records could be beneficial.

### Improvement strategies

The category “Enhancing Record Keeping and Forensic Use” was identified under the theme of improvement strategies.

“I believe strategies like conducting training workshops and digitalizing records can enhance dental record-keeping practices, but stricter laws are needed for its universal implementation.“ (FIR5).

The research participants have argued for a multifaceted approach to improve dental record-keeping practices, suggesting training, digitalization, and stronger regulatory oversight as key components, which aligns with much of the current literature on health information management [37]. The emphasis on training workshops corresponds with research that indicates the importance of proper training in ensuring the successful implementation and adoption of new record-keeping practices. Education can help dental professionals understand the benefits of efficient record-keeping, the risks associated with poor practices, and the functionality of new tools like digital systems [48].

The suggestion to digitalize records supports the growing body of evidence that digital health records can improve efficiency, accessibility, and care coordination. Electronic health records have been shown to increase the accuracy and completeness of documentation, facilitate data retrieval, and enhance the overall quality of care.

Dental records play a critical role in forensic odontology, such as the identification of individuals by comparing antemortem and postmortem dental records, assessment of age and gender, detection of abuse and violence, and legal evidence [12].

### Recommendations

It is crucial for dentists to maintain accurate, complete, and up-to-date dental records. In addition to their primary role in patient care and management, these records can contribute significantly to forensic investigations and justice. Furthermore, stronger laws may be required if these strategies are to be universally adopted. This research demonstrates the importance of regulatory frameworks in health information management for ensuring compliance with record-keeping standards and protecting patient data. To ensure widespread adoption and standardization across the dental profession, training and technology must be backed up by legislative requirements.

We found a discernible gap in engagement and interest regarding forensic odontology within Karachi. The lack of formal educational infrastructure supporting specialized training in this discipline, endorsed by the Pakistan Medical and Dental Council, is one of many reasons for this shortfall. Our study reported that most dentists maintained dental records even in the absence of state laws. Enhanced training and technological advancement might be vital in overcoming these hurdles, enabling dentists to make the most of the valuable information encapsulated in dental records. Further research is warranted to delve into this fascinating intersection of dentistry, record-keeping, and forensic science.

Effective dental record-keeping is essential for patient care, communication, and legal defense, and it demands a meticulous approach. Records should be comprehensive and include all relevant patient information, from demographic details to notes about each visit. Correcting errors transparently and promptly updating any changes is paramount to accuracy. It is also important to record information consistently, and using standardized formats and terminologies can enhance clarity. Entries should be made promptly after each patient interaction to ensure all essential details are captured accurately. Confidentiality must always be maintained, with secure storage for records and access only provided to authorized personnel. The study highlights a lack of standardized dental record-keeping protocols in Pakistan. Developing and implementing such protocols can improve the consistency and comprehensiveness of dental record-keeping across the country. This will facilitate the creation of a unified database for all dental records.

Dental records are currently not required by regulatory bodies in Pakistan. There is a dire need to create awareness for maintaining dental records. Embracing digital record-keeping can increase efficiency and make records easier to access and share, when necessary, but it must be supported by adequate training and strong cybersecurity measures.

There is limited awareness among Pakistani dentists about forensic odontology. Public awareness campaigns could be organized to educate dental professionals about the importance and relevance of forensic odontology and how it relies heavily on well-maintained dental records. In Pakistan, there are only a few fully equipped labs for forensic odontology. Given the potential legal implications of forensic odontology, interdisciplinary cooperation between dental professionals, legal experts, and law enforcement can be fostered. This will not only improve the understanding and use of dental records in legal settings but also emphasize the importance of maintaining a comprehensive dental data repository.

Currently, forensic odontology is not included in the dental curriculum as a separate subject. More emphasis should be placed on teaching forensic odontology and its practical implications. In mass disasters, a comprehensive dental data repository can be instrumental in identifying individuals. As such, developing such a database can be a key component in the country’s disaster management strategy. Laws and regulations may be necessary to enforce standardized dental record-keeping protocols. They can also protect the privacy and rights of individuals whose data is stored in the national repository. Lastly, conducting regular audits of record-keeping practices can identify areas for improvement, contributing to the quality and consistency of records over time. By addressing these implications, Pakistan can make significant strides toward establishing a robust national dental data repository that could play a crucial role in healthcare, legal proceedings, and disaster management.

## Conclusions

An assessment of dental practitioners conducted in this study exposed a suboptimal and, at best, moderate level of understanding and attitude towards the established practices in line with dental forensics. There is potential for improvement if appropriate measures are taken to enforce the study of forensic odontology as a compulsory subject for future students. Moreover, the promotion of this field, with support from local law enforcement agencies and the execution of seminars, could serve to heighten both the appeal of the subject and public consciousness about it.

## Data Availability

The data that support the findings of this study are available from the corresponding author upon reasonable request.
